# Gammaherpesvirus Infections in Cattle in Europe

**DOI:** 10.3390/v13122337

**Published:** 2021-11-23

**Authors:** Giuliana Rosato, Andres Ruiz Subira, Mohammed Al-Saadi, Eleni Michalopoulou, Ranieri Verin, Martina Dettwiler, Heli Nordgren, Koen Chiers, Ernst Groβmann, Kernt Köhler, Michael Suntz, James P. Stewart, Anja Kipar

**Affiliations:** 1 Vetsuisse Faculty, Institute of Veterinary Pathology, University of Zurich, Winterthurerstrasse 268, 8057 Zurich, Switzerland; giuliana.rosato@uzh.ch (G.R.); andres.ruizsubira@uzh.ch (A.R.S.); 2 Centre for Clinical Studies, Vetsuisse Faculty, University of Zurich, 8057 Zurich, Switzerland; 3 Department of Infection Biology and Microbiomes, Institute of Infection, Veterinary and Ecological Sciences, University of Liverpool, Liverpool L3 5RF, UK; mohammed.alsaadi@qu.edu.iq (M.A.-S.); J.P.Stewart@liverpool.ac.uk (J.P.S.); 4 Department of Livestock and One Health, Institute of Infection, Veterinary and Ecological Sciences, University of Liverpool, Neston CH64 7TE, UK; e.michalopoulou@liverpool.ac.uk; 5 Department of Veterinary Pathology and Public Health, Institute of Infection, Veterinary and Ecological Sciences, University of Liverpool, Neston CH64 7TE, UK; ranieri.verin@unipd.it; 6 Department of Infectious Diseases and Pathobiology, Vetsuisse Faculty, Institute of Animal Pathology, University of Bern, Länggassstrasse 122, 3012 Bern, Switzerland; martina.dettwiler@vetsuisse.unibe.ch; 7 Department of Veterinary Biosciences, Faculty of Veterinary Medicine, University of Helsinki, Agnes Sjöbergin katu 2, 00014 Helsinki, Finland; heli.nordgren@helsinki.fi; 8 Department of Pathology, Bacteriology and Poultry Diseases, Faculty of Veterinary Medicine, Ghent University, Salisburylaan 133, 9820 Merelbeke, Belgium; Koen.Chiers@Ugent.be; 9 Aulendorf State Veterinary Diagnostic Centre, Löwenbreitestrasse 18/20, 88326 Aulendorf, Germany; Ernst.Grossmann@stuaau.bwl.de; 10 Faculty of Veterinary Medicine, Institute of Veterinary Pathology, Justus-Liebig-University Giessen, Frankfurter Strasse 96, 35390 Giessen, Germany; Kernt.Koehler@vetmed.uni-giessen.de; 11 State Institute for Chemical and Veterinary Analysis Freiburg, Bissierstrasse 5, 79114 Freiburg im Breisgau, Germany; Michael.Suntz@cvuafr.bwl.de

**Keywords:** BoHV-6, commensal, gammaherpesvirus, qPCR, European cattle

## Abstract

The genus Macavirus, subfamily Gammaherpesvirinae, comprises ungulate viruses that infect domestic and wild ruminants and swine. They cause asymptomatic latent infections in reservoir hosts and malignant catarrhal fever in susceptible species. Lung, spleen, bronchial lymph node, and tongue were collected from 448 cattle (348 necropsied, 100 slaughtered) in Switzerland, United Kingdom, Finland, Belgium, and Germany to determine their infection with bovine herpesvirus-6 (BoHV-6) and gammaherpesviruses of other ruminants, i.e., ovine herpesvirus-1 and -2, caprine herpesvirus-2, and bison lymphotropic herpesvirus, using quantitative PCR. Only BoHV-6 was detected, with an overall frequency of 32%, ranging between 22% and 42% in the different countries. Infection was detected across all ages, from one day after birth, and was positively correlated with age. There was no evidence of an association with specific disease processes. In positive animals, BoHV-6 was detected in all organs with high frequency, consistently in the lungs or spleen. Viral loads varied substantially. In BoHV-6-positive gravid cows, organs of fetuses tested negative for infection, indicating that the virus is not vertically transmitted. Our results confirm previous data indicating that BoHV-6 is a commensal of domestic cattle not associated with disease processes and confirm that infections with other macaviruses are rare and sporadic.

## 1. Introduction

Herpesviruses are enveloped double-stranded DNA viruses and the cause of numerous human and animal diseases. Primary herpesvirus infection generally results in a productive infection that is subsequently limited by the host immune response, leaving behind latently infected cells that persist in the host [1].

Members of the subfamily Gammaherpesvirinae have a narrow host range and cell tropism. They infect lymphocytes, epithelial cells, and endothelial cells [2]; however, latency in B cells is their predominant feature, and some subfamily members cause persistence-associated tumor formation [3]. The genus Macavirus comprises nine ungulate gammaherpesviruses. Macaviruses infect domestic and wild ruminants as well as swine, causing asymptomatic infections in reservoir hosts [4]. However, among these are also the so-called malignant catarrhal fever (MCF) viruses, such as ovine herpesvirus 2 (OvHV-2), alcelaphine herpesvirus 1 (AlHV-1), AlHV-2, and caprine herpesvirus 2 (CpHV-2), that can cause the fatal disease MCF in susceptible species [4].

The reservoir hosts shed virus into the environment and are capable of transmitting it to susceptible hosts when direct or indirect contact occurs, whereas poorly adapted hosts do generally not shed infectious virus and are therefore considered to be dead-end hosts [4]. Other macaviruses, including bovine gammaherpesvirus 6 (BoHV-6) and the porcine lymphocytic herpesviruses (suid herpesvirus (SuHV) 3, 4, and 5), have not been associated with disease, despite being prevalent in cattle and swine [4].

Knowledge on the course of Macavirus infections in reservoir hosts comes from transmission studies using confirmed naturally OvHV-2-infected sheep. These first detected viral DNA in the blood (buffy coat cells), followed by a nasal swab, though at least 7 days later. The virus was also found to be excreted transiently with the semen soon after the blood had turned positive [5], and a later study reports low levels of viral DNA in placenta and amniotic fluids of infected sheep [6]. The latter study also specified that naturally infected sheep shed infectious virus sporadically in nasal secretions, but with only short peaks of large amounts [6]. Experimental OvHV-2 infections of sheep complete the picture and have identified the lung as the site of initial viral replication [7]. After this initial lytic replication, OvHV-2 was primarily found as latent virus in infected peripheral blood leukocytes [7]. Our group has investigated the distribution of OvHV-2 by quantitative PCR in naturally infected sheep and detected the virus with high frequency in lungs and mediastinal lymph nodes (81%) as well as the spleen (91%); it was also present in the tongue with similar frequency (85%), indicating the potential for shedding also from the oral cavity. Interestingly, we also found the virus in these tissues in infected cattle without MCF, though at lower frequency (tongue: 45%; spleen: 38%; lung: 36%; mediastinal lymph node: 30%) and with substantially lower viral loads [8].

BoHV-6, previously known as bovine lymphotropic herpesvirus (BLHV), was first detected and isolated in the USA from a bovine lymphoma induced by bovine leukemia virus (BLV). At that time, its role as a co-factor in BLV-associated disease was proposed [9]. Subsequently, BoHV-6 was identified by PCR in cows suffering from postpartum metritis non-responsive to conventional antibiotic treatment in the UK and other European countries [10,11] and in bovine abortions in Canada [12], suggesting a role in reproductive system disorders. However, experimental data supporting a causative link between BoHV-6 and disease are lacking. In a recent study, BoHV-6 was detected at a low copy number in the brains of two cattle suffering from a non-suppurative encephalitis that was found to be causatively linked to parainfluenza virus 5 infection [13]. Previous molecular studies have shown that BoHV-6 infects peripheral blood mononuclear cells (PBMC) and found infection in 52–87% and 30% of healthy adult cattle and calves, respectively, in the USA and Poland [9,14,15], indicating that BoHV-6 infection is widespread in cattle populations. Infection seems to occur at a very young age; the study in the USA found 38% of the 2-week-old calves were PCR-positive for BoHV-6 [9]. BoHV-6 infection is not exclusive to cattle; viral DNA has been detected in three bison in the USA [14] and seven buffalo (*Bubalus bulalis*) in Brazil [16].

Our group is interested in gammaherpesvirus infections in their natural hosts as opposed to their dead-end hosts and potential protection from infection by related viruses. As part of this, we wanted to assess cattle for infection with BoHV-6 and gammaherpesviruses of other ruminants of which they are not natural hosts, but that can cause MCF in cattle and other ruminants. Accordingly, we examined a large cohort of randomly selected diseased and necropsied as well as slaughtered cattle from several European countries for (co-)infection with the above viruses, using cattle with MCF as “controls” for dead-end host OvHV-2 infection. We tested the lungs, spleen, bronchial lymph node, and tongue, since we had previously found these tissues to carry the virus in OvHV-2 reservoir host infection.

BoHV-6 was identified as the only gammaherpesvirus in all non-MCF cattle, and at high frequency, which prompted us to investigate various population parameters and tissue loads at the individual animal level, in the attempt to better characterize the virus–host interaction and potential effects of BoHV-6.

## 2. Materials and Methods

### 2.1. Animals and Tissue Samples

The study was performed on 448 cattle without MCF that either underwent a diagnostic post-mortem examination after they had been euthanized due to clinical disease or were slaughtered between March 2017 and November 2018. The diagnostic cases had been examined at the Institute of Veterinary Pathology, Vetsuisse Faculty, University of Zurich (*n* = 96); the Institute of Animal Pathology, Vetsuisse Faculty, University of Bern (*n* = 48), Switzerland; the Department of Veterinary Pathology and Public Health, Institute of Infection, Veterinary and Ecological Sciences, University of Liverpool, UK (*n* = 51); the Department of Veterinary Bioscience, Faculty of Veterinary Medicine, University of Helsinki, Finland (*n* = 34); the Department of Pathology, Bacteriology and Poultry Diseases, Faculty of Veterinary Medicine, Ghent University, Belgium (*n* = 50); the Institute of Veterinary Pathology, Justus-Liebig-University Giessen (*n* = 14); the State Institute for Chemical and Veterinary Analysis Freiburg (*n* = 35); and the State Veterinary Diagnostic Centre (Staatliches Tierärztliches Untersuchungsamt) Aulendorf (*n* = 20), Germany. A total of 100 slaughtered animals were sampled at the slaughterhouse in Zurich, Switzerland (*n* = 50), and Wigan, UK (*n* = 50).

From all animals, samples of lung, bronchial lymph node, spleen, and tongue (mucosa and submucosa) were obtained and stored in RNAlater (Sigma-Aldrich, Merck, Darmstadt, Germany) and frozen at −80 °C until processing. In gravid cows (*n* = 3), lungs, bronchial lymph node, spleen, and tongue from the fetus were also collected.

In addition, samples from the same tissues were collected from four necropsied cattle (of which one was gravid) with confirmed OvHV-2-associated malignant catarrhal fever (MCF). These were included as positive controls for the virus and for general comparison of viral loads. A bison that tested positive for bison LHV in lungs, bronchial lymph node, and tongue (with viral loads of 65 copies/100 ng DNA (tongue), 19 copies/100 ng DNA (bronchial lymph node), and 0.5 copies/100 ng DNA (lung)) served as the positive control for bison LHV, and tissues that tested positive for OvHV-1 in a sheep (28,000 copies/100 ng DNA in the lung) and in a cattle (300 copies/100 ng DNA in a lymph node) and for CpHV-2 in a buffalo (22,000 copies/100 ng DNA in the lung) served as the positive control for OvHV-1 and Cp-HV-2, respectively.

For all animals, breed, age, sex, pathological diagnoses, and disease etiology were recorded. For descriptive analyses, animals were grouped into different age ranges. Necropsied animals were grouped according to the primarily affected organ systems, based on recorded pathological diagnoses, as follows: systemic disease and/or multiple organs affected (including, for example, also animals with a diagnosis of cachexia or septicemia), musculoskeletal system, liver, gastrointestinal, cardiovascular system, body cavities (including, for example, animals with a diagnosis of peritonitis due to a perforating foreign body), respiratory system, reproductive system, and other organs affected (including the hemolymphoid systems, the integument, neurological, and urinary system). The latter group was created as the number of cases for each organ system was very low (total of 18 cases). Animals where no abnormality had been detected at necropsy, animals where no further information was available, and the slaughtered animals were each allocated to separate groups. Animals were also grouped according to the disease entity/etiology, creating the following groups: slaughtered animals, unknown entity, traumatic, toxic, congenital, metabolic, bacterial, parasitic, or viral injury/disease, degenerative or neoplastic disease, organ misalignment, or multifactorial disease.

### 2.2. Tissue Processing and DNA Extraction

Tissue samples (25–35 mg) were subjected to DNA extraction with the Qiagen^®^ DNeasy Blood and Tissue kit (Quiagen, Hilden, Germany) according to the manufacturer’s instructions. The eluted DNA was quantified using a NanoDrop^®^ 2000 (Thermo Fischer Scientific Inc., Bartlesville, OK, USA) following the manufacturer’s instructions. The DNA was either used directly or stored at −20 °C until further use. For use in the PCR reactions, the DNA concentration was adjusted to 100 ng genomic DNA.

### 2.3. Quantitative Polymerase Chain Reaction (qPCR)

Quantitative PCR protocols were established for gammaherpesviruses that have previously been shown to infect cattle, i.e., BoHV-6, OvHV-1 and -2, CpHV-2, and Bison LHV [8,15,17].

Primers and probes. The primers for preparation of the standard plasmid DNA were designed manually. For the viruses, conserved sequences within the glycoprotein B (gB) gene were selected as a target for the Taqman qPCR using the GeneScript Bioinformatics software (Genscript Biotech Piscataway Township, NJ, USA; https://www.genscript.com/ssl-bin/app/primer, accessed on 3 February 2015). For OvHV-2, the genome copy number was estimated using OvHV-2 ORF63-specific primers [17], and for BoHV-6, we adopted the published assay based on the gB gene sequence [15]. The possibility of non-specific reactions with other sequences was avoided by using an alignment search tool (BLAST) at the National Centre for Biotechnology Information website (http://www.ncbi.nlm.nih.gov/, accessed on 3 February 2015). The 12s-ribosomal DNA internal genome was used to normalize the qPCR data [18]. Primer and probe sequences as well as gene accession numbers are listed in Table 1. All primers and probes were commercially synthesized (Eurofins Genomics, Wolverhampton, UK).

Optimization of qPCR conditions. The reaction mixtures were optimized to contain 7 μL of (2×) TaqMan^®^ universal PCR master mix (Applied Biosystems; Thermo Fischer Scientific, Warrington, UK); the primers and probes were at a concentration of 0.5 pmol in 20 μL total reaction volume. Initially, the optimal annealing temperatures of primers and probes were determined by gradient protocols (DNA Engine Opticon Biorad, Hertfordshire, UK) in the range of 55–60 °C. The optimal cycling temperature profile in all qPCR assays involved an initial 50 °C for 2 min, followed by 13 min at 95 °C and 39 cycles of 95 °C for 15 s, and, finally, 1 min of the optimal annealing temperature (Table 1).

Production of reference plasmids for viral quantification. The constructed reference genes were produced by cloning the PCR products into the commercial vector pCR2.0 TOPO (Invitrogen) according to the manufacturer’s instructions. The initial constructed plasmid was delivered into one vial of chemically competent Escherichia coli (Mach1™ One Shot^®^, Invitrogen), using the heat-shock method as suggested by the manufacturer. The transformed E. coli was grown to amplify the constructed plasmids that were extracted (Qiagen) and measured using a Qubit fluorimeter (Invitrogen). Normalization to account for variability in DNA quality and contaminants was performed using primers specific for the ruminant 12s-ribosomal DNA internal genome. Therefore, the exact genome copy number and normalization was determined using limiting dilutions of reference plasmids.

Estimation of assay validity. The intra and inter assay variation was analyzed by performing five runs for each reference plasmid in tenfold serial dilutions independently in duplicate starting from 2 × 10^7^ to 2 × 100 copies/2 μL. The assay specificity was determined by cross-testing clinical diagnostic samples known to be positive for other herpesviruses. All quantification statistics were analyzed by 7500 v 2.3 software (Applied Biosystems; Thermo Fischer Scientific) using the Mann–Whitney *t*-test and Fisher’s exact test. The assays were also validated according to MIQE guidelines to include mean value, standard deviation, coefficients of variation (CV) of the Ct values, and the standard error of means for each.

The data regarding the assay’s repeatability and reproducibility were generated from five identical experiments in which the Ct value of duplicates of each dilution of the standard plasmid was calculated. From these data, it was concluded that these assays were highly specific and sensitive hence the assays were used to quantify viral loads.

Testing of animals. In a first step, the lungs of all animals were tested for the ruminant specific 12s-ribosomal DNA internal gene (reference gene for normalization of the data) and for all five selected gammaherpesviruses. Having only identified BoHV-6 in the lung samples of all animals without MCF, the spleen of all animals was tested for ruminant specific 12s-ribosomal DNA, BoHV-6, and OvHV-2, in a second step. In animals that tested positive for BoHV-6 in lungs and/or spleen, bronchial lymph node and tongue were then tested for 12s-ribosomal DNA and BoHV-6.

In cattle with MCF, we applied the same protocol and tested in a first step the lungs for the ruminant-specific 12s-ribosomal DNA and for all five selected gammaherpesviruses. Besides OvHV-2, we detected BoHV-6 in one animal and subsequently tested the remaining organs (spleen, bronchial lymph node, and tongue) for OvHV-2 and BoHV-6. Since the BoHV-6-positive animal was gravid, we also tested the spleen and the lung of the fetus for BoHV-6 and all four organs for OvHV-2. In the remaining BoHV-6-positive gravid cows (*n* = 3), the lungs and spleen of the fetus were tested for the ruminant specific 12s-ribosomal DNA and BoHV-6.

### 2.4. Statistical Analyses

Data were analyzed using R version 4.0.5 (31 March 2021). Initially, data were analyzed with the Shapiro–Wilk test for normal distribution. Logarithmic transformation was applied to viral counts for all organs to achieve a normal distribution and perform parametric analyses (ANOVA and Pearson’s correlation). For the remaining parameters (age, country of origin, etc.), non-parametric analyses were performed using χ2 to examine the association of the parameters with BoHV-6 infection.

## 3. Results

### 3.1. Screening of Cattle for Gammaherpesvirus Infection

Screening of the lungs of all test animals without MCF for OvHV-1, OvHV-2, BoHV-6, Cp-HV-2, and Bison LHV revealed infection with BoHV-6 in 115 samples (26%) but did not find any other viruses tested for. The subsequent testing of all spleens for BoHV-6 and OvHV-2 did again only detect BoHV-6, in 107 animals with a positive result in the lungs, and in 27 animals where the lung was negative for BoHV-6 (total of 134 samples; 30%). These results confirmed BoHV-6 infection in a total of 32% (142/448) of the tested non-MCF animals. In addition, one of the four cattle with MCF was found to be co-infected with BoHV-6.

### 3.2. BoHV-6 Infection

BoHV-6 was detected in cattle from all five contributing countries. Table 2 summarizes the number and age distribution of animals sampled from each country and the origins of the samples (postmortem examination for diagnostic purposes or sampled at slaughter) and whether BoHV-6 was detected in the collected samples.

The probability to detect BoHV-6 infection in an animal was found to be independent of the country where the sample was collected (χ2 = 7.3488; *p* = 0.1186); it was also independent of whether the animal was slaughtered or necropsied (χ2 = 2.7524; *p* = 0.097). However, the case cohort collected in Germany comprised by far the lowest proportion of positive cases (22%), whereas the UK cohort had the highest percentage of positive cases (41%). Given the type of sampling (with and without slaughterhouse material), this cannot be considered a reflection of the prevalence in each of these countries.

**Figure 1:**
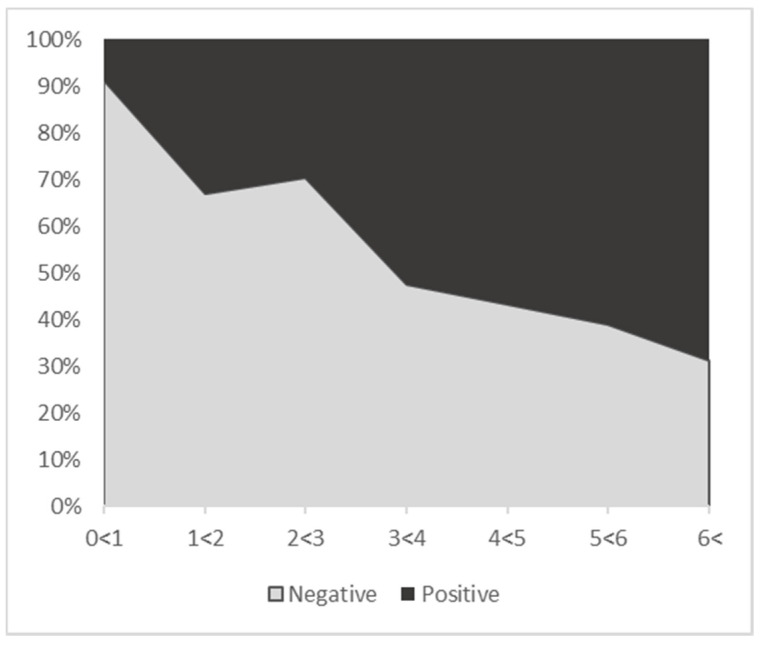
Age distribution of cattle without MCF examined in the present study, with proportions of BoHV-6-positive and -negative animals. Overall, 32% of the tested animals were BoHV-6-positive.

BoHV-6-positive animals ranged in age from 1 day to 13.62 years, with an average age of 3.86 years (Table 2). They were significantly older than negative animals (Mann–Whitney test, *p* < 0.001), which had an average age of 1.47 years (Table 2). For further analysis, animals were allocated by age to seven categories, each spanning one year. Figure 1 summarizes the age distribution for all tested animals and shows that the proportion of positive animals was increasing with age.

BoHV-6 infection was significantly associated with animal sex (χ2 = 9.1496, *p* < 0.001), with female animals infected more frequently (36%) than males (24%) (Supplemental Table S1A). There was a significant association of BoHV-6 infection with the animals’ age groups (χ2 = 95.948; *p* < 2.2e × 10^−16^), shown in 1-year steps up to >6 years of age, with a significant increase in the proportion of infected animals with age. Based on the Chi2 test (Table 3), it was found that in animals younger than 1 year, the probability of BoHV-6 infection was 0.09 lower than expected, with a goodness of fit test of 9.865 and a *p*-value of <0.01. On the other hand, older animals (>6 years) showed a stronger tendency to be positive for BoHV-6 (the probability was 0.04 higher than expected).

Investigation of the association between BoHV-6 infection and both sex and age group showed that, in male animals younger than 1 year, a negative result was substantially more frequent than expected (0.11 expected vs. 0.61 observed) (Supplemental Table S1B).

Investigation for an association between BoHV-6 infection and the primarily affected organ systems (Supplemental Table S2A) found a significant association (χ2 = 25.263; *p* < 0.0042). The correlation line has the best fit for the following organ systems: body cavities, liver, reproductive system, respiratory system, “others”, and “slaughtered animals”. However, there are multiple organ systems involved as well as non-diseased animals, so these apparent associations are likely to be non-specific. There was no significant association between BoHV-6 infection and the disease entity/etiology (Supplemental Table S2B) as identified in diseased animals during the diagnostic postmortem examination (χ2 = 8.87, *p* = 0.7141).

### 3.3. Viral Loads in Different Organs

All animals that tested positive for BoHV-6 in lungs and/or spleen were also tested for the presence of the virus in bronchial lymph nodes (BLN) and tongue. In more than half of the positive cases (*n* = 81; 57%), BoHV-6 DNA was detected in all tested organs. In another 38 cases (28%), three organs were positive. Table 4 summarizes the different combinations of samples where viral DNA was detected.

**Figure 2:**
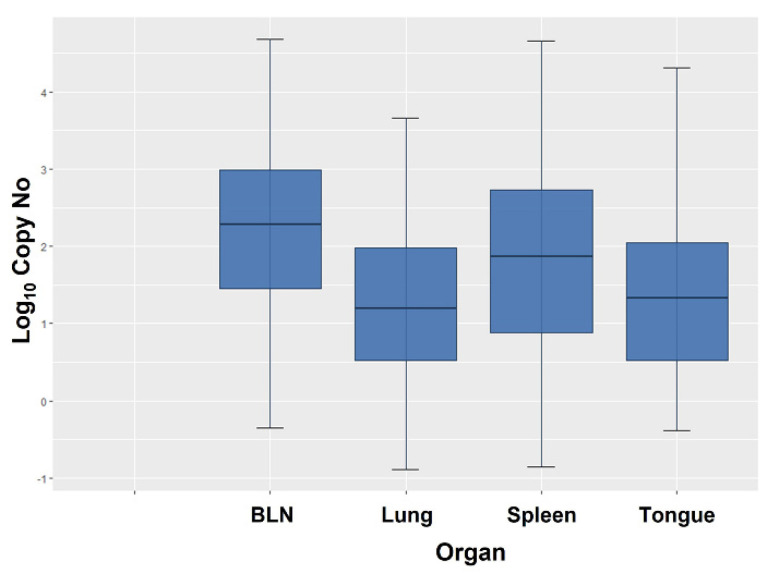
Box plot showing the BoHV-6 Log_10_ copy numbers per 100 ng DNA in each tested organ. Values are expressed as mean ± 2SD, with first quartile (q1), median, and third quartile (q3).

Viral copy numbers varied substantially in the different organs and animals, ranging from as few as 0.13–0.45 copies/100 ng DNA to as many as 47,609 copies/100 ng DNA in a BLN (Figure 2). The variation was highest in BLN and spleen, whereas it was comparatively limited in the lung and tongue (Table 5).

Based on the geometric means obtained from the positive organs, the viral load was overall highest in the BLN, followed by the spleen, the tongue, and the lung (Table 4).

**Figure 3:**
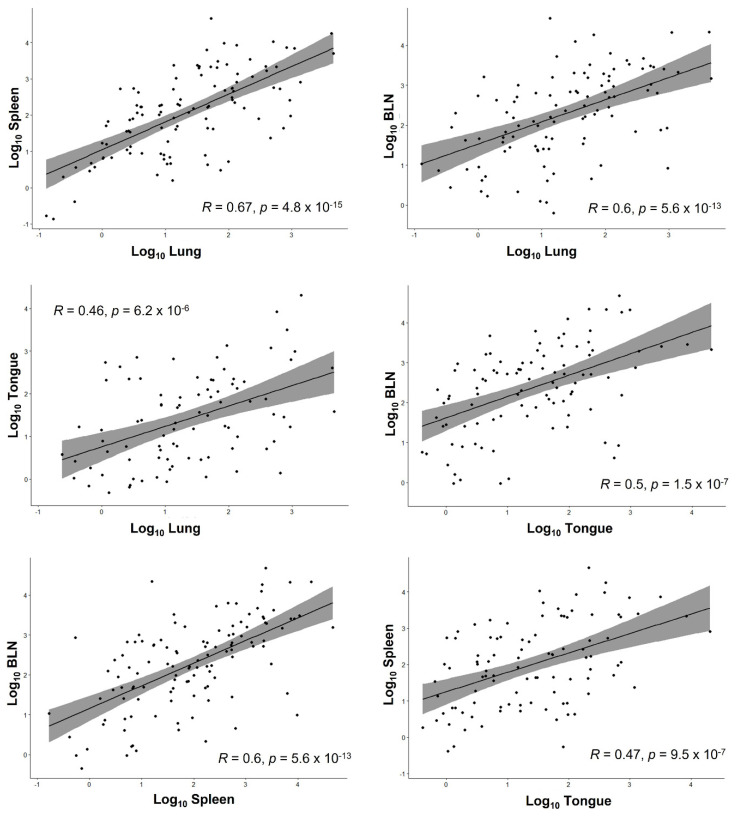
Scatter plots showing the relationship between the Log_10_ copy number for each organ pair with the R square (correlation coefficient) of the regression line, the confidence interval, and the *p*-value following the Pearson test.

Transformation to decimal logarithm served to normalize the data and find associations between the viral load found in the different organs using the Shapiro–Wilk normality test. This did not reveal a significant correlation for viral copy numbers between the different organs (R2 below 0.75). The highest R2 was found between lung and spleen (R2 = 0.67), indicating a high probability to find both organs infected once one was found to be positive. The results are shown in Table 5 and in Figure 3.

Analysis of variance indicated that there was no significant association between the viral loads of any of the tissues tested and the different population parameters (age, sex or country of origin, or disease category/affected organ system).

In the three gravid BoHV-6-positive cows (aged 6, 7, and 12 years), viral copy numbers in the tested organs/tissue were consistently very low (max. 25.2 viral copies/100 ng DNA) and thereby substantially lower than average. In none of the gravid cows was BoHV-6 DNA detected in the lungs and spleen of the fetus.

### 3.4. Cattle with MCF

All animals with confirmed MCF carried OvHV-2 DNA in all tested tissues (lung, BLN, spleen, and tongue), with highly variable viral copy numbers, ranging from less than 1000 copies/100 ng DNA in spleen and BLN in one animal to 77,351 copies/100 ng DNA in the lung in another. One pregnant cow, a 5 year old Braunvieh with MCF, was found to be co-infected with BoHV-6. This animal showed the comparatively highest OvHV-2 copy numbers (per 100 ng DNA) in all tested organs (lung, 77,351; spleen, 36,829; BLN, 32,312; tongue, 10,556) and carried BoHV-6 DNA in all tested organs, though with low viral copy numbers, ranging from 14.63 copies/100 ng DNA in the lung to 529.74 copies/100 ng DNA in the BLN. The organs of the fetus (SSL 49 cm; approx. 5 months of pregnancy) carried OvHV-2 with low viral loads (copies/100 ng DNA): lung 9.52; spleen 4.24; lymph node 2.78; tongue 4.98; and were negative for BoHV-6.

An overall comparison of OvHV-2 loads in the tested organs of cattle with MCF with BoHV-6 loads in the same organs of the non-MCF cattle showed that MCF is associated with significantly higher viral OvHV-2 loads than BoHV-6 infection in cattle (Supplemental Table S3).

## 4. Discussion

In the present study, 448 slaughtered or diseased cattle without MCF were examined for infection with gammaherpesviruses known to infect cattle (OvHV-1, OvHV-2, BoHV-6, CpHV-2, Bison LHV) in tissues that have previously been shown to carry these viruses [8,19], using qPCR. The aim was to gather data on the prevalence of infections and evidence of potential protection of animals infected with one gammaherpesvirus from infection by related viruses.

Taqman q-PCR assays were used to detect and quantify the viruses in question. In the present study, they allowed the detection of very low infection levels, down to 1 copy/100 ng DNA of all viruses tested for.

Interestingly, we only detected BoHV-6, confirming previous studies that cattle are its natural host. The overall prevalence of 32% in our study cohort from countries across Europe is similar to the prevalence previously reported from the USA and Poland [9,14,15]. Although our study is based on a large cohort of cattle from five different countries across Europe and thus also from different forms of husbandry and production, no significant difference was identified between the countries with regards to the rate of BoHV-6 infection. A potential breed predisposition could not be fully investigated due to partially incomplete and highly variable information, but a basic screen did not provide any evidence of the latter (data not shown). However, BoHV-6 infection was significantly associated with age, showing an increasing prevalence in older animals. BoHV-6 infection was also associated with sex, as the percentage of positive animals was significantly higher in female cattle. However, it has to be considered that the older age groups had a lower proportion of male animals.

Previous studies on BoHV-6 infection mainly focused on its association with other infections or diseases. Rovnak et al. [9], who were the first to detect the virus, found viral DNA in blood cells of 94% and 87% of cattle that were seropositive and -negative for BLV, respectively, and in overall 91% of adult cattle and 38% of calves that they had collected from dairy herds in the USA (Colorado, New York, New Jersey) over a 13-year period. Since they also detected the virus in 63% of BLV-positive lymphomas, the authors proposed BoHV-6 as a co-factor in the pathogenesis of BLV and proposed the name bovine lymphotropic herpesvirus [9]. Other studies focused on the association of BoHV-6 with the reproductive tract. Banks et al. [11] examined the vaginal swabs and exudates of cows from 13 herds with a history of post-partum metritis (PPM) that did not respond to standard treatments and detected BoHV-6 in 27% of either sample types. While they considered this to be an indication of a potential role of BoHV-6 in PPM, they were aware that the correlation could be random as they did not examine any PPM-unaffected herds [11]. Similarly, the present study found an association with pathological processes in the reproductive tract. However, these represented variable changes in both BoHV-6-positive animals (mastitis (*n* = 5), dystocia (*n* = 2), each 1 uterine rupture, and spermic granuloma), and BoHV-6-negative animals (mastitis (*n* = 4), metritis (*n* = 2), each 1 metritis and mastitis, retained placenta and endometritis, and uterine rupture). Different from OvHV-2, which we could also detect in fetal tissues in a gravid cow with MCF, BoHV-6 was not detected in the lung or spleen of the fetuses in the four BoHV-6-positive gravid cows in the present study, indicating that the virus is not transmitted transplacentally. There is one previous report that described a case of an aborted fetus in Canada in which BoHV-6 was found in the brain and lymph nodes [12]. However, the fetus was found to also be infected with Neospora caninum, a protozoan parasite leading to abortion in the earlier phase of pregnancy and inducing placental epithelial necrosis, serum leakage, and maternal mononuclear cell infiltration [20]. Destruction of the placental barrier could therefore have allowed BoHV-6 to spread into fetal tissue. However, we found BoHV-6 infection in one of the six 1 day old calves (and, overall, in 3/45 calves aged between 1 and 15 days), which suggests that infection can occur perinatally and/or shortly after birth. However, the prevalence of infection increased with age, and there were no age-associated differences in viral loads.

More general surveys on BoHV-6 prevalence, similar to the present study, have previously been undertaken in the USA. In 2000, Collins and co-authors detected BoHV-6 in blood leukocytes in 52–79% dairy cows in four different herds in Colorado, USA, backing the older study by Rovnak and coworkers [9]. In the present study, the prevalence of BoHV-6 was substantially lower, ranging between 22% and 41% depending on the country. However, the case selection varied significantly, since we examined animals that were either necropsied due to disease or slaughtered, whereas the other studies examined healthy dairy cows or samples collected as part of BLV focused studies [9,14]. Nonetheless, the present study provides clear evidence that BoHV-6 is endemic in cattle across Europe. We found an association with a range of primarily affected organs, not only the reproductive tract as discussed above but also, for example, the respiratory tract or the liver, again with heterogenous processes, and also with being a slaughtered animal. However, it does not indicate association with specific diseases at all. BoHV-6 seems to differ from other gammaherpesviruses, for example, ovine herpesvirus 2, which is very widespread in the domestic sheep population, with more than 90% of animals reported to be persistently infected [4].

Experimental studies using murine gammaherpesvirus-68 (MHV-68) and OvHV-2 in their natural hosts have suggested that gammaherpesviruses initially replicate in the lungs; subsequent latency occurs in lymphoid tissues [21,22]. For BoHV-6, the sites of initial replication and latency have not yet been identified. The present study provides evidence that lungs and lymphatic tissues are involved and confirm the systemic spread of the virus in natural infections.

Further studies are needed to identify the viral target cells in the tissues and to identify the sites of viral persistence/latency. So far, we do not know how BoHV-6 is transmitted, but detection of viral DNA in the tongue where we primarily sampled the epithelium suggests that it might also shed orally, which would be consistent with contact transmission, as known for most herpesviruses.

In contrast to studies on OvHV-2 in their natural host where generally low viral loads were detected [5,19], the present study detected not only highly variable but partly high BoHV-6 loads in tissues, reaching a maximum of more than 47,000 copies per 100 ng DNA. This variability could be due to the heterogeneity of the tested cattle population and a variable length of infection; animals were infected at an unknown time point and could have been exposed to variable initial viral doses.

The study also included a few MCF cases, to compare OvHV-2 loads in diseased animals as dead-end hosts of the virus. Indeed, all MCF cases exhibited higher mean OvHV-2 loads in the tissues than BoHV-6 loads in non-MCF cattle, though the maximum copy number in spleen and BLN were lower. Additionally, the cattle with MCF were single-infected, with one exception, a gravid cow that was also positive for BoHV-6, but with low viral loads. These results are similar to those of a previous study that found BoHV-6 co-infection in 1/18 cattle and 3/21 bison with MCF (spleen and/or lymph node) but observed no significant correlation [14].

Previous studies have detected OvHV-2 in healthy domestic cattle [19,23]. To our surprise, we did not detect OvHV-2 or OvHV-1 in any cattle without MCF in the present study, a result which is in contrast to those of previous studies [8,19,24,25]. This discrepancy is most likely not due to the method of testing, as we applied the highly sensitive quantitative for all viruses that was established in the study of Al-Saadi [8]. An explanation could be that the farmers in Europe are by now well-aware of the risk of MCF due to contact of cattle with sheep and therefore may attempt to prevent the latter, knowing that this could prevent an MCF outbreak in the farm. The fact that MCF cases were rare among the necropsied cattle during the last few years in all diagnostic laboratories contributing to the study supports this interpretation.

Transmission of OvHV-2 to susceptible animals is predominantly horizontal [4], with nasal dissemination being demonstrated experimentally [26,27]. Although reports confirming the vertical transmission of OvHV-2 in cattle are sparse, viral DNA has been detected in an asymptomatic calf born to a cow with sheep associated-MCF [28] and in the brain of the fetus of a cow with MCF [29]. In our study, we detected OvHV-2 DNA in the organs of a fetus from a gravid cow with MCF, which provides further evidence of vertical transmission of OvHV-2 in naturally infected cattle. In contrast to this, we did not find any evidence of vertical transmission of BoHV-6, since all fetuses from BoHV-6-positive gravid cows were negative for BoHV-6. Consequently, horizontal transmission remains the most likely route of BoHV-6 infection to susceptible animals.

## 5. Conclusions

BoHV-6 is the only gammaherpesvirus endemic in European cattle populations with relatively high prevalence. There is no evidence that infection is associated with any disease processes, suggesting that BoHV-6 is a commensal in cattle.

## Figures and Tables

**Table 1:** Taqman primer and probe sequences used for the qPCR.

Target [Accession]	Primer/Probe Sequence 5′→3′	Ann. Temp.
OvHV-1 [D. Griffiths, G. Russell, unpublished]	F: GTATGGCAGCCGTTAGTTCA	57.3 °C
	R: AAGGCGTAGACGCTTCATCT	
	Probe: FAM-CTCGCTTAGCGTCTACAAGCTGTTGC-TAMRA	
BoHV-6 [KJ705001]	F: ACCCCGTAAAAGTGATTTACCC	56.4 °C
	R: GTAGTAGTCATGCATAGCTAGC	
	Probe: FAM-CAAAAGATCAGAGAGCAGCAAGAG-TAMRA	
OvHV-2 [DQ198083]	F: GAGAACAAGCGCTCCCTACTGA	56 °C
	R: CGTCAAGCATCTTCATCTCCAG	
	Probe: FAM-AGTGACTCAGACGATACAGCACGCGACA-TAMRA	
Bison LHV [KX062140]	F: GGTTTGCTTCCCTGCTTAAA	60 °C
	R: CTCCAAGTCTGCGAGCTGTA	
	Probe: FAM-CTTTCCAGCATGGTCCGCCC-TAMRA	
CpHV-2 [NC043059]	F: TCAAGAGCAACAGGAACCAG	60 °C
	R: CTATGCTGCTCACCACGTTT	
	Probe: FAM-AGGCTGCCAAAGGCGTCCAC-TAMRA	
12s-internal DNA [17]	F: GCGGTGCTTTATAYCCTTCTAGAG	60 °C
	R: TTAGCAAGRATTGGTGAGGTTTATC	
	Probe: **VIC**-AGCCTGTTCTATAAYCGAT-**MGBNFQ**	

FAM: 5′ modification (6-Fluorescein amidite); TAMRA: 3′ modification quencher; VIC: 5′ modification: commercial fluorescent dye, MGBNFQ: 5′ modification (minor grove binder nucleotide quencher); Y: (Pyrimidine) C or T; R: (purine) A or G.

**Table 2:** Geographical origin of cattle without MCF examined in the present study and the test results for BoHV-6 infection. Animals were either diseased and had undergone a diagnostic post mortem examination (“Diagnostics”) or were healthy slaughtered cattle (“Slaughterhouse”). Age and sex of the examined cattle are reported regardless of whether they had undergone a diagnostic post mortem examination or were healthy slaughtered cattle. For 5 animals, the sex was not reported, leading to a total of 443 animals considered for the evaluation.

Age Range of the Examined Cattle						
	Switzerland	UK	Finland	Belgium	Germany	Total
**Diagnostics**						
Age range (average)	1 d–12 y (3.1 y)	2 d–6 y (1.46 y)	1 d–10 y (2.7 y)	1 d–10 y (1.63 y)	1 d–10 y (1.2 y)	1 d–13 y (2.23 y)
BoHV-6 pos	43 (30%)	17 (33%)	12 (35%)	16 (32%)	15 (22%)	103 (30%)
BoHV-6 neg	101	34	22	34	54	245
Total tested	144	51	34	50	69	348
**Slaughterhouse**						
Age range (average)	11 mo–13.6 y (2.79 y)	10 mo–2.75 y (1.62 y)				
BoHV-6 pos	15 (30%)	24 (48%)				39 (39%)
BoHV-6 neg	35	26				61
Total tested	50	50				100
**All**						
Age range (average)	11 mo–13.6 y (3.02 y)	10 mo–6 y (1.54 y)	1 d–10 y (2.7 y)	1 d–10 y (1.63 y)	1 d–10 y (1.2 y)	1 d–13 y (2.22 y)
BoHV-6 pos	58 (30%)	41 (41%)	12 (35%)	16 (32%)	15 (22%)	142 (32%)
BoHV-6 neg	136	60	22	34	54	306
**Total tested**	194	101	34	50	69	448
**Age and sex of the examined cattle**						
	**Switzerland**	**UK**	**Finland**	**Belgium**	**Germany**	**Total**
**Age Range (average)**						
BoHV-6 negative	1 d–12 y (2.07 y)	2 d–6 y (1.22 y)	1 d–6 y (1.64 y)	1 d–9 y 11 mo)	1 d–6.5 y (6 mo)	1 d–12 y (1.47 y)
BoHV-6 positive	1 mo–13.6 y (5.24 y)	2 d–6 y (2.00 y)	4 mo–13 y (4.65 y)	1 d–10 y (3.12 y)	1 d–10 y (3.77 y)	1 d–13.6 y (3.86 y)
**Sex**						
Male: positive/tested	10/45 (22%)	15/36 (42%)	1/7 (14%)	4/18 (22%)	3/34 (9%)	33/140 (24%)
Female: positive/tested	48/149 (32%)	26/62 (42%)	11/35 (31%)	12/32 (38%)	12/35 (34%)	109/303 (36%)
**Total: Positive/Tested** **(%)**	58/194 (30%)	41/98 (42%)	12/42 (26%)	16/50 (32%)	15/69 (22%)	142/443 (32%)

Abbreviations: d—days; mo—months; y—years; BoHV-6 pos—PCR-positive for BoHV-6 in at least one tested organ; BoHV-6-neg—PCR negative in all tested organs.

**Table 3:** Frequency distribution of the probabilities for BoHV-6 infection in the different age groups, based on the Chi Square test. Differences (“Expected—Observed”) higher than 0.05 and lower than −0.05 between expected and observed represent an unexpected significant result (highlighted in bold), not following the distribution.

	Expected		Observed		Difference	
Age Group (y)	Neg	Pos	Neg	Pos	Neg	Pos
0 < 1	0.26	0.12	0.35	0.03	**−0.09**	**0.09**
1 < 2	0.15	0.07	0.15	0.07	0.00	0.00
2 < 3	0.07	0.03	0.07	0.03	0.00	0.00
3 < 4	0.06	0.03	0.04	0.05	0.02	−0.02
4 < 5	0.05	0.02	0.03	0.04	0.02	−0.02
5 < 6	0.03	0.01	0.02	0.02	0.01	−0.01
≥6	0.07	0.03	0.03	0.07	0.04	−0.04
All	0.68	0.32	0.68	0.32		

Abbreviations: y—years; neg—PCR for BoHV-6-negative in all tested organs; pos—PCR for BoHV-6-positive in at least one tested organ.

**Table 4:** Frequency of the detection of BoHV-6 in the different tested organs of cattle, alone, in combinations of two or three organs, and in all organs. Numbers and percentages indicate how many of the 142 animals tested positive for the virus were tested positive in the listed combination of organs.

Organs	No of Pos Cases	Percentage
Lung	3	2
Spleen	8	6
Lung + spleen	5	4
Lung + BLN	2	1
Lung + tongue	0	0
Lung + spleen + tongue	4	3
Lung + BLN + tongue	3	2
Lung + spleen + BLN	17	12
Spleen + BLN	5	4
Spleen + tongue	0	0
Spleen + BLN + tongue	14	10
All organs	81	57

Abbreviations: BLN—bronchial lymph node; pos—PCR for BoHV-6-positive in the listed organ(s).

**Table 5:** BoHV-6 copy numbers in the different organs tested by q-PCR. The R square values of the correlation found between the different organs are based on Log10 BoHV-6 copy numbers.

Viral Loads in the Different Organs Tested				
	Lung	Spleen	BLN	Tongue
Median (copies/μL)	15	73	193	22
Average (copies/μL)	196	1232	1873	431
Max (copies/μL)	4558	46,222	47,609	20,441
Min (copies/μL)	0.13	0.14	0.45	0.41
No tested positive	115	134	122	102
% tested positive	81	94	86	72
**R Square Value of the Correlation between the Different Organs**				
**R2**	**Lung**	**Spleen**	**BLN**	**Tongue**
Lung	1			
Spleen	0.67	1		
BLN	0.50	0.60	1	
Tongue	0.46	0.47	0.50	1

Abbreviations: BLN—bronchial lymph node; copies/100 ng—BoHV-6 copy numbers/100 ng DNA; No tested positive—number of animals in which the organ tested positive for BoHV-6 DNA by PCR; % tested positive—percentage of BoHV-6-positive animals in which the organ tested positive.

## Supplementary Materials

The following are available online at https://www.mdpi.com/article/10.3390/v13122337/s1, Table S1: BoHV-6 infection in female and male animals and in the different age groups. Table S2: Frequency of BoHV-6 infection in animals grouped according to the main affected organ system or the disease entity, based on recorded pathological diagnoses. Table S3: Comparison of OvHV-2 loads in lung, spleen, bronchial ln and tongue of cattle with MCF with BoHV-6 loads in the same organs in cattle without MCF.

## References

[1] Roizman B., Pellett P.E., Knipe D.M. (2001). The Family Herpesviridae: A Brief Introduction, Fields–Virology.

[2] Nash A.A., Dutia B.M., Stewart J.P., Davison A.J. (2001). Natural history of murine gammaherpesvirus infection. Philos. Trans. R. Soc. Lond. B Biol. Sci..

[3] Weidner-Glunde M., Kruminis-Kaszkiel E., Savanagoude M. (2020). Herpesviral latency-common themes. Pathogens.

[4] Russell G.C., Stewart J.P., Haig D.M. (2009). Malignant catarrhal fever: A review. Vet. J..

[5] Hüssy D., Janett F., Albini S., Stauber N., Thun R., Ackermann M. (2002). Analysis of the pathogenetic basis for shedding and transmission of ovine gamma herpesvirus 2. J. Clin. Microbiol..

[6] Li H., Taus N.S., Lewis G.S., Kim O., Traul D.L., Crawford T.B. (2004). Shedding of ovine herpesvirus 2 in sheep nasal secretions: The predominant mode for transmission. J. Clin. Microbiol..

[7] Li H., Cunha C.W., Davies C.J., Gailbreath K.L., Knowles D.P., Oaks J.L., Taus N.S. (2008). Ovine herpesvirus 2 replicates initially in the lung of experimentally infected sheep. J. Gen. Virol..

[8] Al-Saadi M.H.A. (2018). Pathogenesis of Malignant Catarrhal Fever in Cattle. Ph.D. Thesis.

[9] Rovnak J., Quackenbush S.L., Reyes R.A., Baines J.D., Parrish C.R., Casey J.W. (1998). Detection of a novel bovine lymphotropic herpesvirus. J. Virol..

[10] Cobb S.P., Banks M., Russell C., Thorne M. (2006). Bovine lymphotrophic herpesvirus in a UK dairy herd. Vet. Rec..

[11] Banks M., Ibata G., Murphy A.M., Frossard J.P., Crawshaw T.R., Twomey D.F. (2008). Bovine lymphotropic herpesvirus and non-responsive post-partum metritis in dairy herds in the UK. Vet. J..

[12] Gagnon C.A., Allam O., Drolet R., Tremblay D. (2010). Quebec: Detection of bovine lymphotropic herpesvirus DNA in tissues of a bovine aborted fetus. Can. Vet. J..

[13] Hierwegen M.M., Werder S., Seuberlich T. (2020). Parainfluenza virus 5 infection in neurological disease and encephalitis of cattle. Int. J. Mol. Sci..

[14] Collins J.K., Bruns C., Vermedah T.L., Schiebel A.L., Jessen M.T., Schultheiss P.C., Anderson G.M., Dinsmore R.P., Callan R.J., DeMartin J.C. (2000). Malignant catarrhal fever: Polymerase chain reaction survey for ovine herpesvirus 2 and other persistent herpesvirus and retrovirus infections of dairy cattle and bison. J. Vet. Diagn Investig..

[15] Kubiś P., Materniak M., Kuźmak J. (2013). Comparison of nested PCR and qPCR for the detection and quantitation of BoHV6 DNA. J. Virol. Methods.

[16] de Oliveira C.H.S., de Oliveira F.G., Gasparini M.R., Galinari G.C.F., Lima G.K., Fonseca A.A., Barbosa J.D., Barbosa-Stancioli E.F., Leite R.C., dos Reis J.K.P. (2015). Bovine herpesvirus 6 in buffaloes (*Bubalus bulalis*) from the Amazon region, Brazil. Trop. Anim. Health Prod..

[17] Stahel A.B.J., Baggenstos R., Engels M., Friess M., Ackermann M. (2013). Two different macaviruses, ovine herpesvirus-2 and caprine herpesvirus-2, behave differently in water buffaloes than in cattle or in their respective reservoir species. PLoS ONE.

[18] Gatesy J., Amato G., Vrba E., Schaller G., DeSalle R. (1997). A cladistic analysis of mitochondrial ribosomal DNA from the Bovidae. Mol. Phylogenet. Evol..

[19] Amin D. (2015). Infection of the Ovine Herpesvirus 2 in the Reservoir Host, Sheep, and the Susceptible Host, Cattle. Ph.D. Thesis.

[20] Gibney E.H., Kipar A., Rosbottom A., Guy C.S., Smith R.F., Hetzel U., Trees A.J., Williams D.J.L. (2008). The extent of parasite-associated necrosis in the placenta and foetal tissues of cattle following *Neospora caninum* infection in early and late gestation correlates with foetal death. Int. J. Parasitol..

[21] Hughes D.J., Kipar A., Sample J.T., Stewart J.P. (2010). Pathogenesis of a model gammaherpesvirus in a natural host. J. Virol..

[22] Taus N.S., Schneider D.A., Oaks J.L., Yan H., Gailbraith K.L., Knowles D.P., Li H. (2010). Sheep (*Ovis aries*) airway epithelial cells support ovine herpesvirus 2 lytic replication in vivo. Vet. Microbiol..

[23] Powers J.G., VanMetre D.C., Collins J.K., Dinsmore R.P., Carman J., Patterson G., Brahmbhatt D., Callan R.J. (2005). Evaluation of ovine herpesvirus type 2 infections, as detected by competitive inhibition ELISA and polymerase chain reaction assay, in dairy cattle without clinical signs of malignant catarrhal fever. J. Am. Vet. Med. Assoc..

[24] Yazici Z., Arslan H.H., Gumusova S.O., Meral Y., Albayrak H. (2006). Occurrence of ovine herpesvirus type-2 infection in sheep and cattle in Samsun Province, Turkey. Dtsch Tierarztl. Wochenschr..

[25] Kojouri G.A., Mahmoodi P., Momtaz H. (2009). Identification of SA-MCFV DNA in blood, lymph node, and spleen of adult sheep, healthy cattle, and MCF cattle by PCR. Comp. Clin. Pathol..

[26] Li H., Snowder G., O’Toole D., Crawford T.B. (1998). Transmission of ovine herpesvirus 2 in lambs. J. Clin. Microbiol..

[27] Nishimori T., Ishihara R., Kanno T., Jayawardane G.L., Nishimori K., Uchida I., Imai K. (2004). Experimental transmission of ovine herpesvirus-2 in sheep. J. Vet. Med. Sci..

[28] O’Toole D., Li H., Miller D., Williams W.R., Crawford T.B. (1997). Chronic and recovered cases of sheep-associated malignant catarrhal fever in cattle. Vet. Rec..

[29] Headley S.A., Pimentel L.A., Oliveira V.H.S., Toma H.S., Alfieri A.F., Carvalho A.M., dos Santos M.D., Alfieri A.A. (2015). Transplacental transmission of ovine herpesvirus 2 in cattle with sheep-associated malignant catarrhal fever. J. Comp. Pathol..

