# Supplementary material for: Gammaherpesvirus Infections in Cattle in Europe
Source: Viruses. 2021 Nov 23;13(12):2337. doi: 10.3390/v13122337 (PMC8709109; doi:10.3390/v13122337)
Supplement: Supplementary file 1 [file viruses-13-02337-s001.zip › viruses-1377411-supplementary.pdf]

viruses

MDPI

Article

Gammaherpesvirus infections in cattle in Europe

Table S1. BoHV-6 infection in female and male animals and in the different age groups. The sex was not known for 5 of the 448 examined animals. The frequency of the probabilities for BoHV-6 infection in the different age and sex groups is based on the Chi2 test. Differences ("Expected – Observed") higher than 0.05 and lower than -0.05 between expected and observed (in bold) represent an unexpected result, not following the distribution.

BoHV-6 Infection in Male and Female Animals			
**Sex**	**Neg**	**Pos**	**All**
Male	107	33	140
Female	194	109	303
Total	306	142	443

Frequency of The Probabilities for BoHV-6 Infection								
	**Male**				**Female**			
**Age Group (y)**	**Expected**		**Observed**		**Expected**		**Observed**	
	**Neg**	**Pos**	**Neg**	**Pos**	**Neg**	**Pos**	**Neg**	**Pos**
0 <1	0.11	0.01	**0.61**	0.06	0.24	0.02	0.22	0.02
1 <2	0.05	0.02	**0.12**	0.13	0.10	0.05	0.16	0.05
2 <3	0.02	0.01	0.01	0.03	0.05	0.02	0.10	0.03
3 <4	0.01	0.01	0.00	0.01	0.03	0.03	0.06	0.07
4 <5	0.01	0.01	0.01	0.01	0.02	0.03	0.04	0.05
5 <6	0.00	0.01	0.00	0.00	0.01	0.02	0.02	0.04
≥6	0.01	0.02	0.01	0.00	0.02	0.05	0.05	0.11

neg – PCR for BoHV-6 negative in all tested organs; pos – PCR for BoHV-6 positive in at least one tested organ; y – years.

Table S2. Frequency of BoHV-6 infection in animals grouped according to the main affected organ system or the disease entity, based on recorded pathological diagnoses.

Grouping According to the Primarily Affected Organ Systems			
**Affected Organ System**	**Neg**	**Pos**	**All**
No abnormality detected	6	0	6
No information available	16	2	18
Systemic disease and/or multiple organs affected	36	9	45
Musculoskeletal	18	9	27
Liver	9	7	16
Gastrointestinal	85	23	108
Cardiovascular	11	4	15
Body cavities	8	9	17
Respiratory	39	21	60
Reproductive	9	9	18
Others	8	10	18
Slaughtered animals	61	39	100
Total	306	142	448
**Grouping According to Disease Entity or Aetiology**			
**Disease Entity/Etiology**	**Neg**	**Pos**	**All**
Slaughtered animals	67	39	106

Unknown	43	13	56
Trauma	23	11	34
Congenital	5	2	7
Metabolic	6	6	12
Degenerative	2	2	4
Toxic	2		2
Bacterial	105	48	153
Parasitic	11	5	16
Viral	10	2	12
Neoplastic	2	2	4
Organ misalignment	15	7	22
Multifactorial	15	5	20
Total	306	142	448

Others: Due to the low number of cases, animals which showed primary affection of the haemolymphatic, neurological or urinary system or the integument were assembled under this heading. Abbreviations: y – years; neg – PCR for BoHV-6 negative in all tested organs; pos – PCR for BoHV-6 positive in at least one tested organ.

Table S3. Comparison of OvHV-2 loads in lung, spleen, bronchial ln and tongue of cattle with MCF with BoHV-6 loads in the same organs in cattle without MCF.

**Viral Loads**	**Lung**		**Spleen**		**BLN**		**Tongue**	
	**MCF (OvHV-2)**	**Non-MCF (BoHV-6)**	**MCF (OvHV-2)**	**Non-MCF (BoHV-6)**	**MCF (OvHV-2)**	**Non-MCF (BoHV-6)**	**MCF (OvHV-2)**	**Non-MCF (BoHV-6)**
Median (copies/100 ng)	10,063.46	15.35	13,749.81	72.67	4,430.70	193.08	17,320.12	21.58
Average (copies/100 ng)	25,135.96	195.66	16,294.11	1,232.27	10,530.52	1,873.49	19,235.23	431.10
Max (copies/100 ng)	77,350.72	4,558.00	36,829.21	46,222.00	32,312.40	47,609.00	37,262.72	20,441.00
Min (copies/100 ng)	3,066.22	0.13	847.59	0.14	948.26	0.45	5,037.91	0.41
No tested positive	4	115	4	134	4	122	4	102

Abbreviations: copies/100 ng – BoHV-6 or OvHV-2 copy numbers/100 ng DNA; No tested positive – number of animals in which the organ tested positive for BoHV-6/OvHV-2 DNA by PCR.
